# Metformin: Activation of 5′ AMP-activated protein kinase and its emerging potential beyond anti-hyperglycemic action

**DOI:** 10.3389/fgene.2022.1022739

**Published:** 2022-10-31

**Authors:** Sanjay Goel, Ravinder Singh, Varinder Singh, Harmanjit Singh, Pratima Kumari, Hitesh Chopra, Rohit Sharma, Eugenie Nepovimova, Martin Valis, Kamil Kuca, Talha Bin Emran

**Affiliations:** ^1^ Government Medical College, Patiala, Punjab, India; ^2^ Chitkara College of Pharmacy, Chitkara University, Punjab, India; ^3^ Department of Pharmacology, Government Medical College and Hospital, Chandigarh, India; ^4^ Department of Rasa Shastra and Bhaishajya Kalpana, Faculty of Ayurveda, Institute of Medical Sciences, Banaras Hindu University, Varanasi, Uttar Pradesh, India; ^5^ Department of Chemistry, Faculty of Science, University of Hradec Králové, Hradec Králové, Czechia; ^6^ Neurology Clinic, University Hospital, Hradec Králové, Czechia; ^7^ Department of Neurology, Charles University in Prague, Faculty of Medicine in Hradec Králové and University Hospital, Hradec Králové, Czechia; ^8^ Andalusian Research Institute in Data Science and Computational Intelligence (DaSCI), University of Granada, Granada, Spain; ^9^ Department of Pharmacy, BGC Trust University Bangladesh, Chittagong, Bangladesh; ^10^ Department of Pharmacy, Faculty of Allied Health Sciences, Daffodil International University, Dhaka, Bangladesh

**Keywords:** hyperglycemia, oxidative stress, cardioprotective, anticancer, metformin

## Abstract

Metformin is a plant-based drug belonging to the class of biguanides and is known to treat type-2 diabetes mellitus (T2DM). The drug, combined with controlling blood glucose levels, improves the body’s response to insulin. In addition, trials have identified the cardioprotective potential of metformin in the diabetic population receiving the drug. Activation of 5′ AMP-activated protein kinase (AMPK) is the major pathway for these potential beneficial effects of metformin. Historically, much emphasis has been placed on the potential indications of metformin beyond its anti-diabetic use. This review aims to appraise other potential uses of metformin primarily mediated by the activation of AMPK. We also discuss various mechanisms, other than AMPK activation, by which metformin could produce beneficial effects for different conditions. Databases including PubMed/MEDLINE and Embase were searched for literature relevant to the review’s objective. Reports from both research and review articles were considered. We found that metformin has diverse effects on the human body systems. It has been shown to exert anti-inflammatory, antioxidant, cardioprotective, metabolic, neuroprotective, anti-cancer, and antimicrobial effects and has now even been identified as effective against SARS-CoV-2. Above all, the AMPK pathway has been recognized as responsible for metformin’s efficiency and effectiveness. Owing to its extensive potential, it has the capability to become a part of treatment regimens for diseases apart from T2DM.

## 1 Introduction

Metformin, a biguanide derivative, has been used primarily for managing hyperglycemia by restricting intestinal glucose absorption and decreasing hepatic gluconeogenesis (Wang et al., 2017; Zhao et al., 2020). It is isolated from a traditional European medicinal plant, *Galega officinalis* L. (Fabaceae). Members of the biguanide class were first synthesized in 1920s, and some of them have also been employed for managing type-2 diabetes mellitus (T2DM). Later, in the 1940s, metformin was researched for the purpose of treating malaria (Bailey, 2017; Foretz et al., 2019), was observed to cure influenza (Sharma et al., 2020), and was additionally found to be useful in lowering blood glucose levels (Vera et al., 2019). Jean Sterne, a famous French clinical pharmacologist, further studied this last property of biguanides and reported metformin as an anti-diabetic agent (Bailey, 2017).

Chemically, metformin contains two methyl groups attached to the biguanide segment and is also identified as metiguanide or 1,1-dimethylbiguanide. It exists in white crystalline powder form with hygroscopic properties (Wang et al., 2017).

**FIGURE 1:**
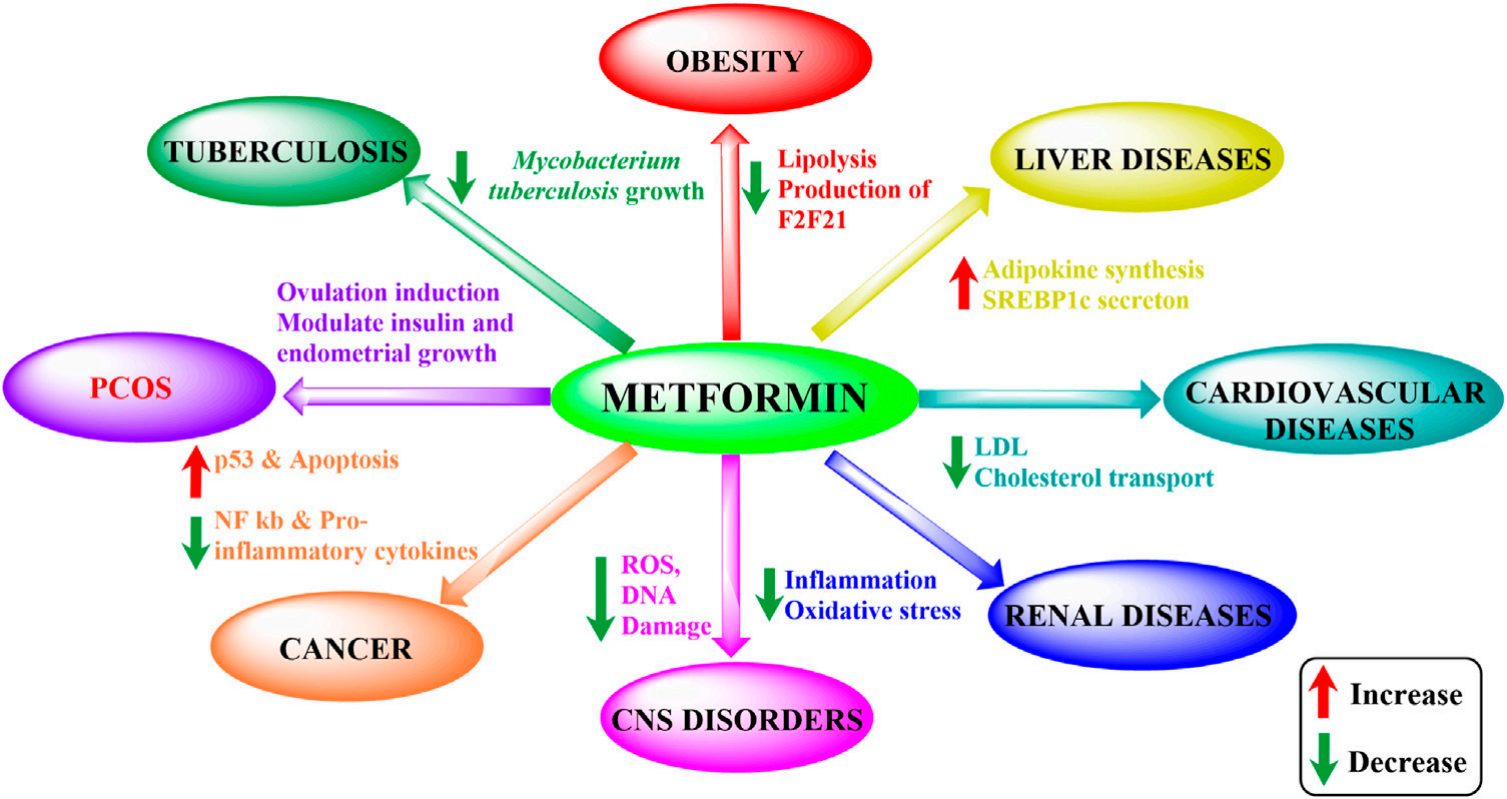
Metformin’s impact on different disorders. ROS, reactive oxidative species; PCOS, polycystic ovary syndrome; LDL, low-density lipoprotein; NF-κB, nuclear factor kappa-light-chain-enhancer of activated B cells; SREBP1c, sterol regulatory element-binding protein 1c).

Metformin is the first-line drug for T2DM but also holds potential for various other conditions (Figure 1). Effects beyond its antihyperglycemic action, such as anti-inflammatory, anti-oxidant, neuroprotective, cardioprotective, anti-microbial, and anti-cancer properties, have been observed in series of studies (Table 1). These effects arose unexpectedly but have surely been proved to be effective to different degrees. Moreover, metformin’s strong effects have also been seen in liver and renal diseases (Mather et al., 2001; Wu et al., 2008; Abdelgadir et al., 2017; Aljofan and Riethmacher, 2019; Lv and Guo, 2020; Shurrab and Arafa, 2020).

**TABLE 1:** Reported studies of metformin in the treatment of different diseases.

SL. No.	Type of study	Study subjects	Dose and duration	Results	References
1	Randomized, prospective, double-blind, placebo-controlled, multicenter study	140 children (boys-72, girls-68) of age: 7 to 14 with BMI >95th percentiles. 2 groups were formed: first group—pre-pubertal and pubertal children—given metformin. Second group—Pre-pubertal and Pubertal children—given placebo	Initially, subjects were asked to take 50 mg BD for first 10 days, followed by 500 mg BD until the end. Total duration: 6 months	BMI score was decreased. Cardiovascular and inflammatory-associated obesity parameters were found to be improved	Pastor-Villaescusa et al. (2017)
2	Controlled clinical trial	154 people with BMI ≥27 kg/m^2^ were chosen for metformin administration. 45 people were taken as control	Metformin 2,500 mg/day. Total duration: 6 months	Metformin was found to be effective in both insulin-resistant and sensitive overweight and obese subjects. In the study group, weight loss was reported by 5.8 ± 7.0 kg. In control group, weight gain was observed by 0.8 ± 3.5 kg	Seifarth et al. (2013)
3	Randomized, double-blind placebo-controlled crossover study	20 non-diabetic patients (of age: 55–80) with Alzheimer’s disease	The subjects were divided into 2 groups, randomly in 1:1 ratio to receive metformin and placebo, respectively. They were asked to begin with 500 mg/day metformin or placebo for a week, gradually the dose was increased to a maximum dose of 2000 mg/day by increasing 500 mg per week. Total duration: 8 weeks	Memory, learning, and executive functioning were found enhanced at the end of the trial	Koenig et al. (2017)
4	Randomized control trial	129 women of age 40–65 were divided into two groups. Control group was treated with chemotherapy + hormonal therapy while the Interventional group received chemotherapy + hormonal therapy + metformin along with vitamin B12 i.m. to both groups every 3 days	The trial began with 850 mg OD for 2 weeks and increased to 850 mg BD within food	Metformin added to the adjuvant breast cancer therapy decreased metastasis and the inducible metastatic factors, HOMAIR and FBG, along with the reduced insulin levels. Increase in IGFBP-3 protective factor and decreased IGF-1 were reported	El-haggar et al. (2016)
5	Controlled clinical trial	11 non-diabetic-newly diagnosed and untreated patients with Endometrial cancer were chosen for the trial	500 mg TID to the subjects for a mean of 36.6 days, considering follow up of mean of 57 months	Anti-proliferative effects of metformin were observed with significant reduction in: IGFBP-7 and IGF-1 Mean plasma insulin Expression of pS6 and ki-67	Laskov et al. (2014)
6	Pre-clinical study	8-week-old non-diabetic non-alcoholic steatohepatitis mice with liver pathologies	37.5 mg/kg of mouse weight (∼2250 mg/60 kg of human) Total duration: 8 weeks	At the end of the trial	Kita et al. (2012)
				• Downregulation in activation of hepatic stellate cells	
				• Suppression in inflammation, fatty acid metabolism and fibrogenesis	
				• Decrease in hepatic triglyceride levels were reported	
				Amelioration in induced inflammation, abnormal retention of lipids-steatosis and fibrosis was seen	

5′ AMP-activated protein kinase activation has been found to be a common link between the many activities of metformin (Agius et al., 2020; Lu et al., 2021). AMPK, adenosine monophosphate-activated protein kinase, is an enzyme which plays a substantial role in maintaining cellular energy homeostasis. It functions when ATP levels fall and influences the uptake of fatty acids and glucose (Garcia and Shaw, 2017). The enzyme is known to be a major regulator in numerous metabolic pathways and hence holds the potential to serve as a constructive therapeutic target for different chronic metabolic diseases. The activation of AMPK brings out various actions mediated in discrete ways, as briefly accounted in Table 2 (Gongol et al., 2013; Day et al., 2017).

**TABLE 2:** Actions of metformin through AMPK activation.

SL. no.	Action	Effects precipitated by AMPK activation	References
1	Anti-inflammatory	Dissociates PARP-1 from BCL-6 intron additionally, increase BCL-6’s expression	Gongol et al. (2013), Cameron et al. (2016)
2	Anti-oxidant	Increases expression of thioredoxin	Apostolova et al. (2020)
3	Cardio-protective	Prevents the opening of mPTP	Driver et al. (2018)
4	Weight loss	Downregulation of hormone—ghrelin	Gagnon et al. (2012)
5	Effective in PCOS	Targets TNF-α	Kai et al. (2015)
6	Neuro-protective	Modulates autophagy	Wang et al. (2016), Sportelli et al. (2020)
7	Anti-cancer	Downregulates GF receptor levels	Lei et al. (2017)
8	COVID-19	Anti-viral (inhibits multiplication, and maturation, inhibit translation of viral proteins, regulate viral protein–host protein interactions) and modulates inflammation and the immune response in COVID-19	Sharma et al. (2020), Samuel et al. (2021)

BCL-6: B-cell lymphoma, mPTP: mitochondrial permeability transition pore, TNF-α: tumor necrosis factor-α, GF: growth factor, PCOS: polycystic ovary syndrome, PARP-1: poly (ADP-ribose) polymerase 1.

This review aims to appraise other potential uses of metformin beyond its anti-hyperglycemic effect. We searched databases including MEDLINE, PubMed, and Embase to locate both research and review articles. Additionally, other resources like DrugBank and certain awareness surveys were searched.

## 2 5′AMP-activated protein kinase and its biological functions

**FIGURE 2:**
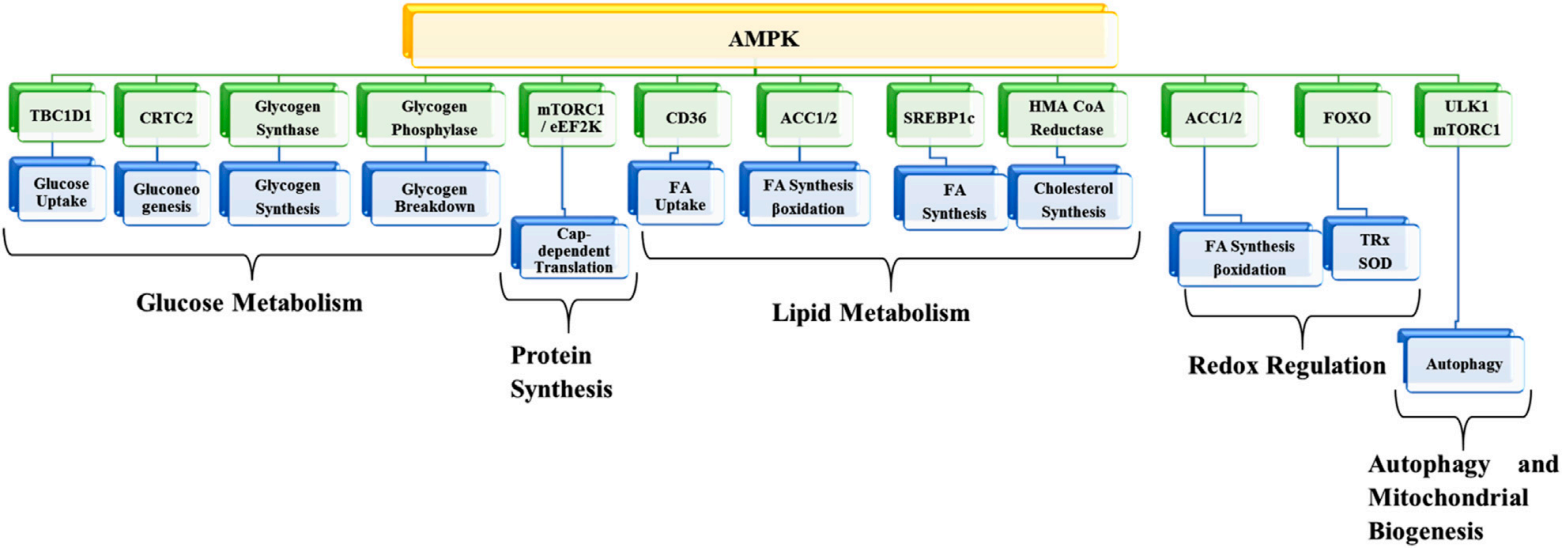
Metabolic functions of AMPK. TBC1D1, TBC1 domain family member 1; CRTC2, CREB-regulated transcription coactivator 2; mTORC1, mammalian target of rapamycin-complex 1; EEF2K, eukaryotic elongation factor 2 kinase; ACC1, acetyl-CoA carboxylase 1; SREBF1, sterol regulatory element-binding transcription factor 1; HMG-CoA, 3-hydroxy-3-methylglutaryl coenzyme A; FOXO, forkhead box transcription factor; ULK1, Unc-51-like kinase 1; FA, Fatty acid; SOD, superoxide dismutase; TRX, thioredoxin.

AMPK, a member of the transferases family, belongs to the class of serine/threonine kinases, which has become one of the most promising potential targets for preventing and treating different kinds of diseases. The enzyme works as a metabolic checkpoint; it is activated following a dip in intracellular ATP levels (normal concentrations of ATP, ADP, and AMP are approximately 1850 µM, 145 µM, and 5 µM, respectively). An 8% percent drop in ATP (i.e., 1710 µM) and a 4-fold increase in AMP (i.e., 20 µM) leads to AMPK activation (Garcia and Shaw, 2017). As a result, metabolic adaption is facilitated by inhibiting anabolic ATP-consuming pathways such as protein synthesis and fatty acid synthesis and by promoting different catabolic ATP-generating pathways like glycolysis, glucose uptake, and fatty acid oxidation. AMPK exhibits numerous biological, metabolic, and physiological functions (Srivastava et al., 2012; Jeon, 2016; Ponnusamy et al., 2020; Wu and Zou, 2020; Trefts and Shaw, 2021) (Figure 2).

## 3 Physiological functions

The physiological consequences of AMPK activity are vast, occurring in response to numerous, multifactorial pathways. AMPK activity mediates mechanisms leading to favorable physiological effects. In the hypothalamus, the anti-obesity hormone leptin inhibits AMPK, suppressing appetite and preventing overeating (Minokoshi et al., 2002; Jeon, 2016). AMPK also targets another anti-obesity hormone, ciliary neurotrophic factor (CNTF), a member of the cytokine family. It suppresses appetite and activates peripheral β-oxidation. Moreover, in leptin-resistant muscles, CNTF differentially regulates AMPK and tends to exhibit physiological effects (Jeon, 2016). There is evidence that AMPK exerts potent anti-inflammatory effects by switching the metabolism of immune cells from glycolysis to mitochondrial oxidative metabolism, while lymphocytes rely only on mitochondrial oxidative metabolism (O’Neill and Hardie, 2013). AMPK and β-oxidation have also been observed to switch pro-inflammatory M1 macrophages into anti-inflammatory M2 macrophages. Interestingly, exercise-induced muscle contraction exhibits insulin-sensitizing effects along with other health-promoting effects, largely via AMPK activation (Jeon, 2016).

## 4 Metabolic functions

### 4.1 Lipid metabolism

Regulation of lipid metabolism is the best-known function of AMPK. AMPK inhibits the synthesis of fatty acids (FAs), triglycerides (TGs), and cholesterol and facilitates β-oxidation and the uptake of Fas. *De novo* FA synthesis is inhibited by the induction of the inhibitory phosphorylation of acetyl-CoA carboxylase 1 (ACC1), which catalyzes the rate-limiting step in FA synthesis, and by sterol regulatory element-binding protein 1c (SREBP1c), a promoting transcription factor of multiple lipogenic enzymes (Li U et al., 2011). AMPK also inhibits glycerol-3-phosphate acyltransferase, which is responsible for catalyzing TG synthesis. Other than these, 3-hydroxy-3-methylglutaryl coenzyme A (HMG-CoA) reductase, an enzyme that is an essential rate-limiting protein in cholesterol biosynthesis, is also inhibited by AMPK-led inhibitory phosphorylation, which eventually limits cholesterol production (Hardie and Pan, 2002; Li Y et al., 2011; Jeon, 2016). Lipid catabolism and anabolism are also activated by AMPK. FA uptake is influenced by AMPK-regulated translocation of CD36 (cluster of differentiation 36), which transports FAs to the plasma membrane, from where they move into the mitochondria for β-oxidation with the help of the enzyme carnitine palmitoyltransferase-1 (CPT-1). AMPK enhances both β-oxidation and CPT-1 activity via the inhibitory phosphorylation of ACC2. Conclusively, AMPK is essential in maintaining the concentration of free FAs by limiting lipolysis and lipogenesis and activating β-oxidation (Daval et al., 2005; Jeon, 2016).

### 4.2 Glucose metabolism

Glucose transporter type 4 (GLUT4) is an insulin-regulated glucose transporter predominantly found in striated skeletal and cardiac muscles and in adipose tissues. Upon activation of AMPK, GLUT4-containing intracellular vesicles are translocated across the plasma membrane. Rab-family G proteins are required, in their active GTP-bound state, to facilitate the fusion of these vesicles with the plasma membrane. AMPK induces inhibitory phosphorylation of TBC1D1, a Rab-GTPase activator, eventually enhancing the activity of Rab-family G proteins and fusion activity. Another glucose transporter, glucose transporter 1 (GLUT1), is regulated by the AMPK-led suppression of thioredoxin interacting protein (TXNIP), causing the phosphorylation and consequent rapid degradation of TXNIP and, ultimately, elevated GLUT1 function. AMPK also increases the mRNA expression of GLUT4- and hexokinase 2-encoded genes (Wu et al., 2013). Moreover, it regulates the glycolysis and glycogenesis processes. AMPK phosphorylates and inhibits glycogen synthase enzyme, restricting glycogen synthesis (Hunter et al., 2011). Glycogen breakdown is also facilitated via AMPK-mediated inhibitory phosphorylation of glycogen phosphorylase. For the regulation of blood glucose levels, hepatic gluconeogenesis is crucially important. AMPK inhibits CREB-regulated transcription coactivator 2 (CRTC2) and hepatocyte nuclear factor 4 (HNF4), like other several-expression of gluconeogenic enzymes promoting transcription factors, which in consequence, inhibit gluconeogenesis (Jeon, 2016).

### 4.3 Protein synthesis

AMPK inhibits the growth regulator mammalian target of rapamycin, or mTOR complex 1 (mTORC1), in consequence of phosphorylation of TSC2 and regulatory-associated protein of mTOR (raptor). mTORC1 inhibition activates a multifunctional protein, eukaryotic translation initiation factor 4E-binding protein 1 (4E-BP1), and inhibits a protein synthesis-inducing enzyme, P70-S6 kinase, leading to inhibition of cap-dependent translation during the initiation process in ribosomal proteins. Translational elongation is inhibited by the activatory phosphorylation of eukaryotic elongation factor 2 (eEF2) kinase, which inactivates eEF2 directly. Moreover, ribosomal RNA synthesis is downregulated by inhibition of AMPK-induced transcription initiation factor 1A (Leprivier et al., 2013; Jeon, 2016).

### 4.4 Redox regulation

AMPK regulates the defense against oxidative stress through both short- and long-term effects. For managing oxidative stress, in antioxidant defense regulation, AMPK plays a role in upregulating various antioxidants, such as superoxide dismutase (SOD), and in uncoupling protein 2 (UP2)-encoded genes. This activates forkhead transcription factor (FOXO), a key protein in redox signaling, by decreasing levels of superoxide and thioredoxin (Trx) (Greer et al., 2007). Another potential AMPK target is NRF2 protein, which regulates the expression of antioxidant proteins via these regulatory pathways and leads to redox regulation (Naveira et al., 2011). AMPK-led inhibitory phosphorylation of ACC1 and ACC2 also contributes to maintaining NADPH levels (Jeon, 2016).

### 4.5 Autophagy and mitochondrial biogenesis

AMPK facilitates autophagy by directly and indirectly targeting Unc-51-like kinase-1 (ULK1). ULK1 is directly phosphorylated and activated by AMPK, leading to autophagy induction. AMPK also activates indirectly by inhibiting mTORC1, leading to ULK1 inhibition, which disrupts ULK1–AMPK interaction. This well-coordinated regulation between ULK1 and mTORC1 helps eliminate damaged mitochondria and attain mitochondrial integrity during nutrient starvation (Egan et al., 2011; Jeon, 2016). AMPK-mediated FOXO activation also contributes to the upregulation of proteins such as B-cell leukemia/lymphoma 2 protein (BCL2) and adenovirus E1B 19-kDa-interacting protein 3 (Bnip3), autophagy-related 12 protein, and microtubule-associated protein 1A/1B-light chain 3 (LC3), which induces autophagy. Autophagy provides substrates for mitochondrial metabolism and supplies energy generation, making mitochondrial biogenesis a crucial part of nutrient deficiency. A variety of pathways regulate the mitochondrial biogenesis stimulating-cofactor peroxisome proliferator-activated receptor-gamma coactivator 1 (PGC1) alpha. This cofactor enhances the transcription of mitochondrial gene transcripts encoded in the nucleus (Jeon, 2016).

## 5 Biological activities of metformin

### 5.1 Anti-inflammatory activity

**FIGURE 3:**
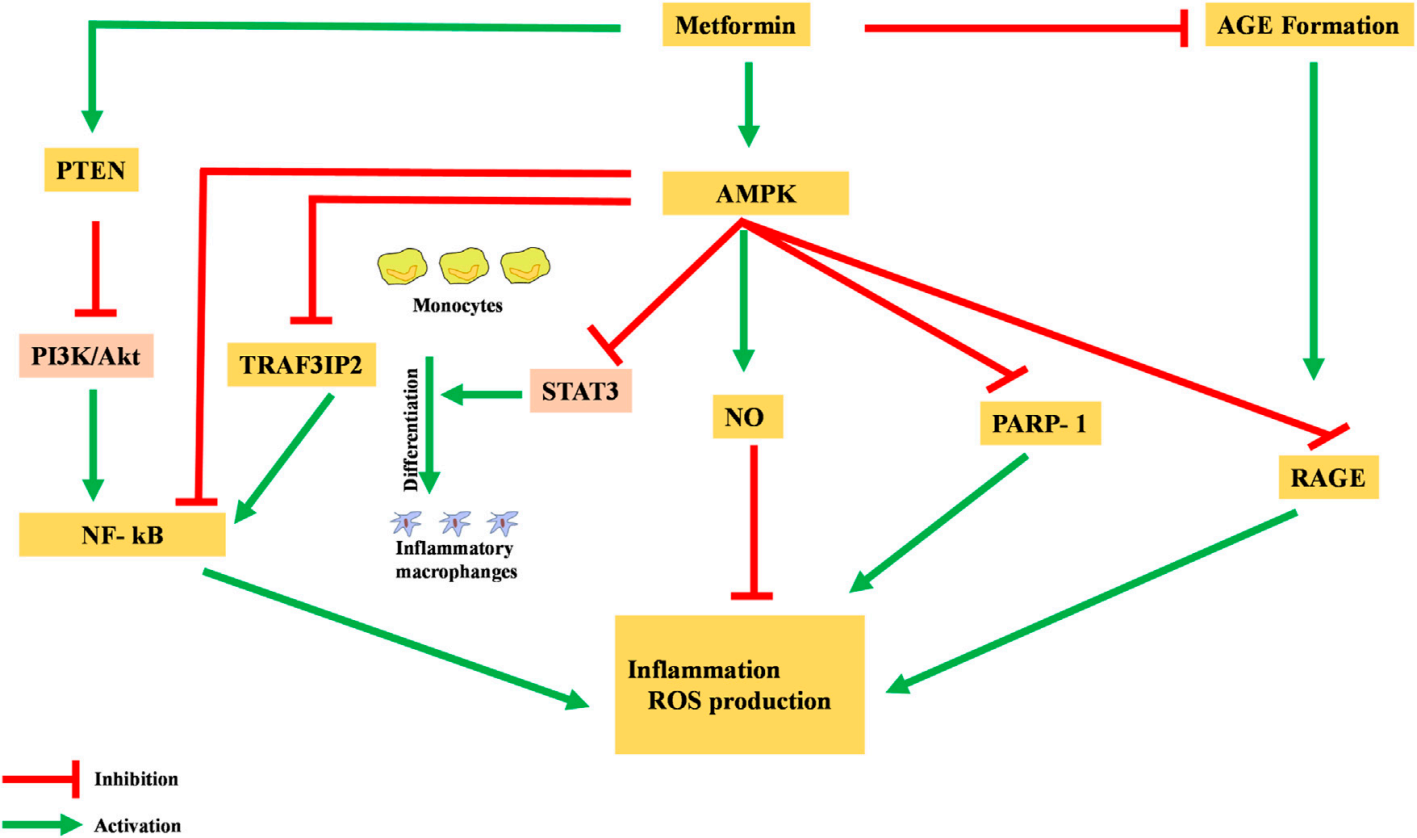
Anti-inflammatory action of metformin via different pathways. NF-κB, nuclear factor kappa-light-chain-enhancer of activated B cells; AGE, advanced glycation end products; ROS, reactive oxidative species.

Metformin has been shown to possess an anti-inflammatory action, mainly via the AMPK-activation-mediated inhibition of a transcription factor, nuclear factor kappa-light-chain-enhancer of activated B cells (NF-κB) (Deng et al., 2020). AMPK activation induces the transcription of specific pro-inflammatory genes, including PARP-1 and BCL6. AMPK activation dissociates PARP-1, which is an NF-κB activator, from the BCL-6 intron and also increases the expression of the *BCL6* gene and its anti-inflammatory action (Figure 3) (Gongol et al., 2013).

Moreover, metformin is reported to inhibit the production of advanced glycation end products (AGEs), which have been observed to promote oxidative stress and inflammation. In macrophages, AGEs bind to receptors for advanced glycation end products (RAGEs), enhance expression of TNF-α, IL-1, and IL-6, and activate the NF-κB pathway, causing inflammation, apoptosis, and fibrotic reactions. Metformin reacts with AGE’s dicarbonyl precursors and inhibits their production. Furthermore, metformin binds to RAGE by AMPK activation and downregulates AGE formation. This binding of metformin to RAGE modulates the phenotypes of surface markers from inflammatory to anti-inflammatory (Saisho, 2015).

Pre-clinical studies in rats have identified the AMPK-phosphatase and tensin homolog (AMPK-PTEN) pathway as part of the restriction of inflammation in smooth muscle cells (Kim and Choi, 2012). In a clinical study, blockage of the phosphoinositol 3 kinase–protein kinase B (PI3K–PKB) pathway was theorized to inhibit activation and action of NF-κB in human vascular smooth muscle cells. Other cells were also reported to have decreased their formation of pro-inflammatory cytokines like TNF and IL-6, as well as PGE2 and NO (Isoda et al., 2005).

### 5.2 Anti-oxidant activity

Metformin has been observed to regulate oxidative balance in various studies by inhibiting the complex I enzyme in the electron transport chain (ETC) (Fontaine, 2018; Vial et al., 2019; Wang et al., 2019). Reactive oxygen species (ROS) production is directly restricted by inhibiting mitochondrial complex I and modulating the respiratory chain (Sanz et al., 2021). Upon metformin administration, ROS production is reduced and an enhanced anti-oxidant system is witnessed (Ouslimani et al., 2005; Diniz et al., 2016). In animals, metformin is reported to increase levels of endogenous proteins such as thioredoxin (Trx), sirtuin 3, and glutathione to counteract the attack of free radicals produced during variety of disorders, including age-related neurodegeneration, epilepsy (Sanz et al., 2021), cardiovascular disease, cancer, inflammation, and osteoporosis. The AMPK pathway increases the expression of Trx, an oxidoreductase enzyme. It tends to attenuate the amount of ROS-mediated metabolic stress by serving as a primary key target for defense (Li et al., 2009). Expression of Trx is upregulated through an Akt-induced transcription factor, forkhead transcription factor 3 (FOXO3). The forkhead-type factors encode for various extracellular and intracellular antioxidant enzymes and proteins like SOD, which tend to effectively control oxidative balance (Birben et al., 2012).

Additionally, levels of the NRF2 protein, which regulates antioxidant expression, were found to be elevated in ischemic rats treated with metformin, along with improved activities of catalase and glutathione (Ashabi et al., 2015).

### 5.3 Cardioprotective activity

Benefits for cardiovascular conditions upon metformin treatment have been identified by many studies reporting lower chances of morbidity and mortality, including in the United Kingdom Prospective Diabetes Study (UKPDS) conducted in the 1990s (King et al., 1999). Gradually, studies have found more evidence for the cardioprotective nature of metformin (Han et al., 2019; Slater et al., 2019). A study by Mohan et al. (2019) highlighted metformin’s potential to improve left ventricular mass index (LVMI) and systolic blood pressure (SBP), as compared to the placebo administered for the experiment. The study involved 68 study subjects with coronary artery disease without type-2 diabetes, aged between 18 and 85 years, who were given 1,000 mg twice daily for 11 months. At the end of the randomized control trial, the group administered with metformin was found to have decreased LVMI along with reduced subcutaneous abdominal tissue and net body weight, indicating the drug’s capacity to improve hypertrophy of the LV. Along with this, thiobarbituric acid-reactive substances and lipid peroxidation byproducts were also reduced, leading researchers to conclude that the decreased oxidative stress resulted from the drug treatment (Mohan et al., 2019).

**FIGURE 4:**
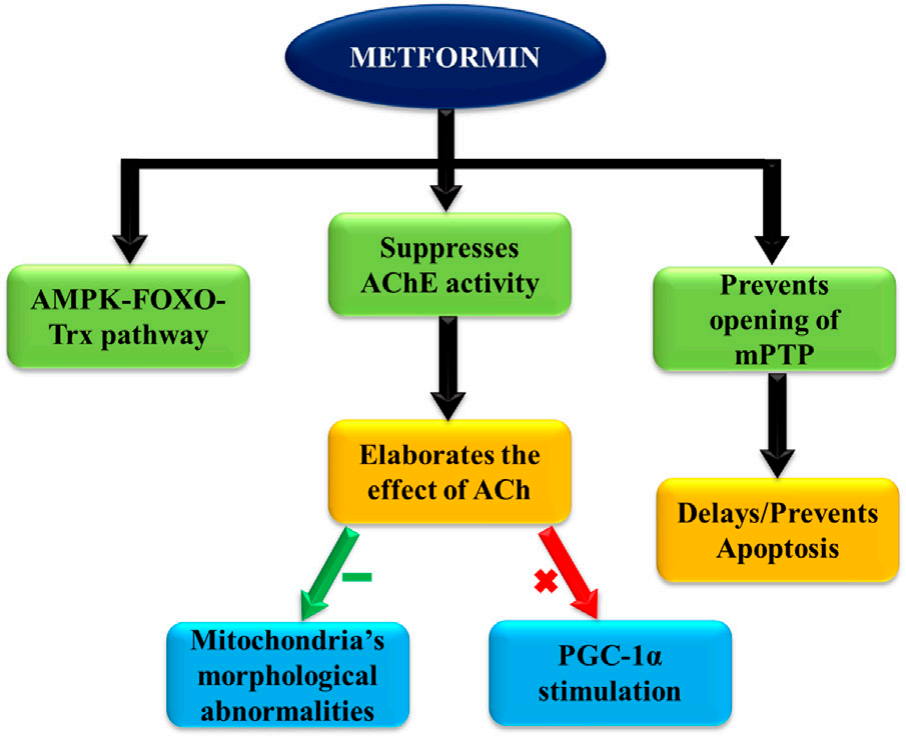
Key actions of metformin contributing to cardioprotective effects.

One of the cardioprotective mechanisms involved is the AMPK-FOXO-Trx pathway, as explained above (Diniz et al., 2016). The improved antioxidant defense attenuates associated pathogenic processes like lipid peroxidation, impaired cardiomyocyte metabolism, and endothelial dysfunction, preventing a number of cardiovascular abnormalities (Figure 4) (Senoner and Dichtl, 2019).

Acetylcholinesterase (AChE) activity is found to be suppressed by metformin administration, which directly improves acetylcholine (Ach) levels. Consequently, ACh significantly inhibits morphological abnormalities of mitochondria, along with stimulating PGC-1α, a transcriptional coactivator. PGC-1α serves as an important factor in mitochondrial biogenesis regulation, which is directly linked with cardiac functioning (Sun et al., 2013).

Moreover, under conditions of myocardial infarction (MI) and ensuing reperfusion, metformin is found to keep the mitochondrial permeability transition pore closed via activating the AMPK pathway, preventing associated cell death in affected tissues. This is reported to reduce the infarct size in study subject animals with acute ischemic reperfusion injury. Henceforth, better and faster recovery and survival rates of patients with CV disorders are expected with metformin use.

### 5.4 Weight loss

A variety of evidence has emerged in support of metformin’s use for weight loss in obese patients. Suggested mechanisms potentially involve major modulation in appetite and the AMPK pathway (Yerevanian and Soukas, 2019). Metformin’s impact on appetite results from both direct and indirect means. Inhibition of ETC’s complex I enzyme and subsequent lactate production has been found to mediate appetite suppression by lactate-induced metabolic acidosis (Islam et al., 2017). In addition, the gut-brain axis and reduction in the orexigenic hormone ghrelin are also reported to play significant roles. Ghrelin hormone is also downregulated through AMPK activation (Gagnon et al., 2012; Yerevanian and Soukas, 2019). Moreover, AMPK activation reduces the production of hepatic glucose. Metformin administration has been associated with enhanced uptake of peripheral glucose in muscle and adipose tissue and with reduced glucose absorption in the intestine and decreased glucose production in the liver, leaving less glucose to be converted into fat molecules. The insulin-sensitizing effect further facilitates weight loss by improving glycemic control. In contrast, many studies of metformin in patients with T2DM have not found a loss in weight but have rather identified metformin as being weight neutral (Bosi 2009; Cheng and Kashyap, 2011). Metformin’s role in weight loss remains debatable owing to its being weight neutral in a few studies as well as weight reduction effects. A clinical trial performed by Seifarth et al. (2013) with 154 non-diabetic, obese study subjects saw significant weight loss of about 5.8 ± 7.0 kg (5.6 ± 6.5%) in the group receiving 2,500 mg/day metformin in comparison to 45 control subjects who instead gained 0.8 ± 3.5 kg (0.8 ± 3.7%). The patients with insulin resistance lost even more than the others (Seifarth et al., 2013). A systematic review performed by Brufani et al. (2013) showed that metformin administration for 6–12 months could moderately lower the weight of obese children and reduce BMI by 1.1–2.7 compared to placebo groups. Studies have spoken for such effects, but further studies are required for more solid and significant conclusions demonstrating weight loss with this drug.

### 5.5 Polycystic ovary syndrome (PCOS) and pregnancy

A meta-analysis performed by Lashen (2010) justified metformin as an efficient option for treatment, finding it to be a first-line candidate for PCOS patients as it initiated effects by inducing ovulation (Lashen, 2010). AMPK activation-mediated action is considered to be the basic mechanism for metformin’s effect (Will et al., 2012). It tends to act primarily by targeting TNF-α, a major ovary-function modulator mediated GROα and IL-8 production and signal transduction pathways in granulosa cells that are seen to be non-apoptotic in PCOS patients. Furthermore, TNF-α suppression inhibits theca-interstitial (T-I) cells of the ovary, countering T-I hyperplasia or hyperthecosis and consequently leading to hyperandrogenism (Convery and Brawer, 1991; Kai et al., 2015).

Additionally, females with PCOS tend to have a higher risk of miscarriages in pregnancy than those without PCOS; the drug is observed to restrict miscarriage risk as well (Lashen, 2010). Upon its use, improvements in perifollicular blood flow and vascularization, uterine blood flow, and ovarian artery impedance are reported, facilitating increased chances of successful pregnancy (Palomba et al., 2009).

### 5.6 Neuroprotective effect

Neurodegeneration progresses via oxidative stress, protein hyperphosphorylation, and the resulting neuro-inflammation. Metformin has been shown to counter these processes by stimulating the antioxidant system and protein dephosphorylation and causing decreased neuro-inflammation (Rotermund et al., 2018; Kulkarni et al., 2020). Evidence suggests that the AMPK pathway is substantially responsible for the neuroprotective effect. AMPK activation modulates neuro-inflammation and autophagy where required. mTOR signaling and neurodegeneration are known to have a co-relation, especially in immune-inflammatory-neurodegenerative disorders. In cases of diverse genetic mutations and acquired abnormalities, mTOR has been a part of pathways ultimately leading to seizures. AMPK pathway activation inhibits the mTOR system (Citraro et al., 2016; LaMoia and Shulman, 2021), which reduces the generation of pro-inflammatory mediators including iNOS, TNF-α, and IL-1β, which is found to be beneficial in providing neuroprotection in the middle cerebral artery occlusion (MCAO) model of cerebral stroke and epilepsy. Metformin has also been shown to provide both symptomatic relief and reduced seizure severity in studies, suggesting its potential for preventing and managing epilepsy (Yimer et al., 2019). Reduced touch response, finger snap, and pick up, and improved cognitive deterioration like ameliorated behavioral manifestations, have been seen with metformin treatment. Metformin also manages seizure severity parameters like duration of seizure experience, seizure score, and mortality, while also increasing latency for the first onset of seizure. All of this contributes to not only longevity but also to a better quality of life. Moreover, metformin prevents rudimentary cellular changes contributing to epileptogenesis, including neuronal cell loss, gliosis, and apoptosis, which are primarily seen in epilepsy. Epileptogenesis progression is strongly promoted by molecular alterations like oxidative stress, and metformin’s ability to deter these increases its anti-epileptic effect (Yimer et al., 2019). The generation and expression of microglia-mediated pro-inflammatory cytokines are also reduced with metformin (Paudel et al., 2020). Additionally, the drug is reported to delay programmed apoptosis by restricting the permeability transition pore cycle and downregulating the permeability transition protein, which is somewhat responsible for initiating apoptotic processes (Rotermund et al., 2018).

Also, the activated AMPK pathway regulates GLUT1 translocation to the plasma membrane, enhancing glucose uptake and glycolysis and preserving neuronal-metabolic functionality (Sanz et al., 2021). In Alzheimer’s and Parkinson’s diseases, alpha-synuclein protein (SNCA) accumulates within the Lewy bodies of neural tissues. Metformin use in patients affected with such disorders has attenuated SNCA levels and prevented its phosphorylation to gradually improve their condition (Xu et al., 2015; Paudel et al., 2020). Metformin also helps in relieving the cognitive alterations, memory, and learning deficits caused by neurodegenerative conditions.

### 5.7 Anti-cancer activity

As in other conditions, activation of the AMPK pathway via activation of liver kinase B1 (LKB1) brings about anti-carcinoma action with metformin. This activation mediates the downregulation of growth factor (GF) receptor levels and the induction of apoptosis following cell cycle arrest (Zheng et al., 2012). Metformin’s anti-cancer mechanisms also involve autophagy and apoptosis by p21, which is supported by p53 family proteins (Vancura et al., 2018; Yi et al., 2019; Podhorecka, 2021).

**FIGURE 5:**
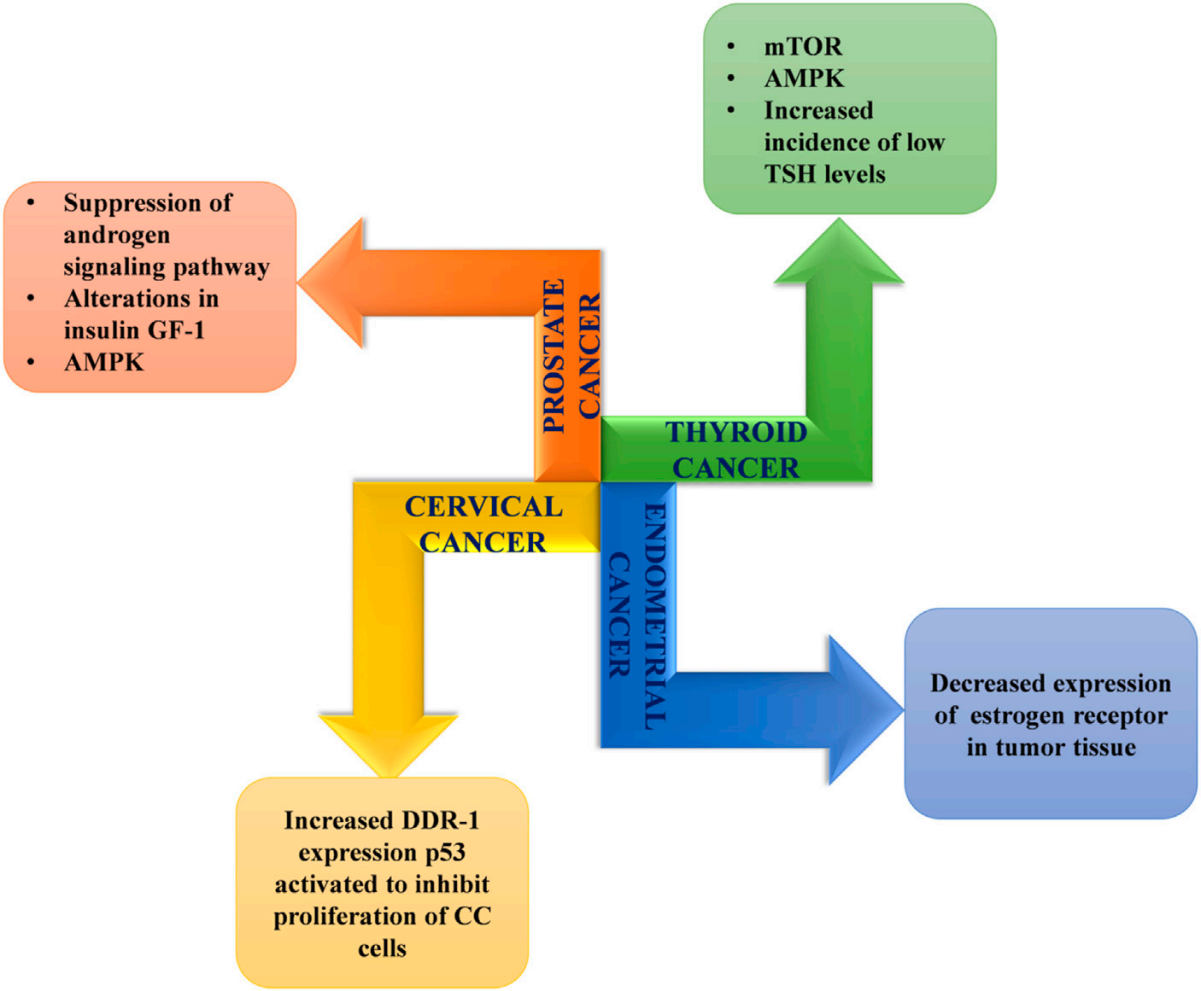
Mechanisms associated with anticancer effects of metformin.

Another associated action is through LKB1-AMPK activation-induced mTOR inhibition. mTOR (mammalian target of rapamycin) is a central regulator of various physiological and metabolic processes and greatly influences cell growth and division (Zheng et al., 2012; Saraei et al., 2019; Guarnaccia et al., 2021). Its inhibition in turn downregulates cell proliferation and cancer development (Figure 5). Moreover, evidence suggests involvement of mTOR and hypoxia-inducible factor 1 (HIF-1) in hypoxia and cancer progression, respectively. Consequently, indirect HIF-1 inhibition also plays a partial role (Greijer and Van-der Wall, 2004; Saraei et al., 2019).

### 5.8 Anti-microbial activity

Metformin has a range of effects, and studies from recent years have now identified its antimicrobial ability as well. The drug’s antimicrobial activity is widely attributed to its ability to activate the AMPK pathway. Its potential against the parasite *Plasmodium falciparum* and influenza virus has been recognized and applied, but it is now being explored for use against other microorganisms as well. Metformin is found to be effective against a number of Gram-negative and Gram-positive bacterial strains, along with a few fungi, parasites, and certain viruses (Masadeh et al., 2021).

Against bacteria and mycobacteria, metformin works primarily through AMPK activation, which greatly contributes to its increased ROS production, indirectly bringing about bactericidal action (Yu et al., 2019). Strains that are known to be affected following ROS production are *Legionella pneumophila*, *Acinetobacter baumannii*, and *Mycobacterium tuberculosis*; in *E. coli*, inflammasome activation leading to increased pyroptosis plays an additional role (Singhal et al., 2014; Liang et al., 2016; Kajiwara et al., 2018). In order to tackle antibiotic resistance, metformin has been used as an adjuvant drug for conventional antibiotics (Patil et al., 2019). It is reported to accelerate and potentiate the effectiveness of antibiotics by working on a person’s immune response (Silwal et al., 2018; Masadeh et al., 2021).

A combination of antifungal agents and metformin is reported to have better fungicidal action than the antifungal drugs alone. Lowered MIC_50_ values have been seen with their concomitant use. The antifungal potential of metformin has been linked with the mTOR–complex 1 pathway (Loos et al., 2020). Metformin and other biguanides inhibit mitochondrial complex I activity, which in turn disturbs the ATP:AMP ratio, lowering ATP and increasing AMP. The ATP-deprived condition prompts changes in the conformation of the nuclear pore complex, while the accumulation of AMP inhibits mTORC1. It is one of the important growth regulator pathways, which when halted conveniently works against fungal strains (Xu L et al., 2018).

A study by Meherunisa et al. (2018) assessing the antimicrobial properties of metformin saw dose-dependent activity against microorganisms, with commendable results in the average zone of inhibition of 12–15 mm at the dose with concentration 500 µg/µl. Zone diameters in the bacterial strains of *E. coli* and *P. aeruginosa* with metformin were 15 mm and 13 mm, respectively, in comparison to the antibiotic amikacin (30 µg), which displayed a bactericidal effect with a diameter of 16 mm and 14 mm against *E. coli* and *P. aeruginosa*, respectively. Zone diameters of 14 mm and 17 mm were observed in the fungal strains of *Candida albicans* , respectively (Meherunisa et al., 2018).

Metformin’s antiviral effects have been reported in both DNA and RNA viruses. In addition to AMPK pathway activation, metformin counters cytokine-mediated damage, significantly reducing severity. The drug has been found to be active against viruses including dengue virus, coxsackievirus B3 (CVB3), Kaposi’s sarcoma-associated herpesvirus, and hepatitis B virus, along with SARS-CoV-2 (Chenet al., 2020). ACE-2 receptor, the host receptor for SARS-CoV-2, is used for viral entry into the cells. Upon AMPK activation, metformin interferes with the interaction between the virus and the ACE-2 receptor (Chen et al., 2020; Davidson et al., 2020). Moreover, suitable combinations, like metformin combined with antiviral drugs and others, can be researched and established, making the formulation more effective as a whole (Yu et al., 2012; Chen et al., 2018; Chen et al., 2020).

### 5.9 Sepsis

Sepsis occurs primarily due to the body’s altered response to infections and involves the dysregulation of anti-inflammatory and pro-inflammatory responses (Rudd et al., 2020). Its incidence is increasing while awareness and seriousness about the condition among the public remains extremely low; progress in its treatment options is also low in comparison to the severity and potential of the disease. Gradual but steep is the increasing rate of mortality of patients with sepsis, the majority of which cases are attributed to multi-drug resistance, demonstrating the need for new alternatives and combinations (Zilberberg et al., 2014; Busani et al., 2019; Gandra et al., 2019). Metformin’s immunosuppressant property is seen to be beneficial for the condition. A study by Tsoyi et al. (2011) has concluded that metformin use increases the rate of survival in patients. The drug LPS, which is known to initiate an inflammatory response against pathogens, was observed to ameliorate the expression of pro-inflammatory cytokines like COX-2, inducible nitric oxide synthase, and their mediators generated by the latter induced response (Kim et al., 2007; Tsoyi et al., 2011; Malik et al., 2018).

High-mobility group box protein 1 (HMGB1), a cytokine, has been known to serve as a therapeutic target for years. It is known to have the potential to cause further damages by initiating a systemic inflammatory response. Metformin is considered to inhibit its release substantially (Tsoyi et al., 2011; Yang et al., 2020). As an additional unlikely effect, antibiotics are also observed to maintain gut permeability and prominently increase beneficial gut bacteria. Metformin’s ability to attenuate inflammatory mediators and prevent thrombus formation suggests a capacity for other actions as well (Malik et al., 2018).

### 5.10 Tuberculosis

Tuberculosis (TB), caused by bacteria *Mycobacterium tuberculosis*, is a menace throughout the world*.* Treatments for TB have benefited from reasonable amounts of advances and successes, but are still held back by certain limitations of the strain, which is drug-resistant and has long-term treatment requirements. The use of metformin in conjunction with conventional antibiotics has been shown to have potential as an alternative for managing TB (Patil et al., 2019). The drug exerts an inhibitory effect on mitochondrial complex I, which subsequently alters the AMP:ATP ratio. This generated altered cellular energy environment activates the AMPK pathway. The bacterial killing is precipitated via the stimulation of endothelial nitric oxide synthase following AMPK activation (Padmapriyadarsini et al., 2019). In macrophages, metformin also fosters phagocytosis, phagolysosome fusion, and autophagy. After metformin exposure, macrophages exhibit greater bactericidal capacity owing to their increased mitochondrial ROS, accelerating the fusion of the phagosome and lysosome, which promotes subsequent bacterial death. Moreover, metformin controls immunopathology and promotes the immune response, which also contributes to improvement (Tseng, 2018; Padmapriyadarsini et al., 2019; Patil et al., 2019).

Adding metformin to regimens during these times of increasing antibiotic resistance can surely be beneficial in enhancing immunity and early killing of the pathogen, as has been shown in certain studies involving both diabetic and non-diabetic subjects (Padmapriyadarsini et al., 2019). The underlying reason to accommodate metformin remains its ability to diminish inflammation and to control subsequent lung tissue damage and *Mycobacterium* growth, but the number of studies with unconvincing and debatable results for metformin use in such conditions has required caution during use, as well as more experimentation and the establishment of a proper regimen (Malik et al., 2018; Padmapriyadarsini et al., 2019).

Other than these, the drug has also been found to be effective against *Staphylococcus aureus*, *Trichinella spiralis*, human immunodeficiency virus, and hepatitis C virus (Malik et al., 2018). Effectiveness with good safety profiles has been witnessed against pathogens, though more studies and better research results are certainly required for putting metformin’s properties to use in conventional therapies.

## 6 Potential role of metformin in COVID-19

Metformin has been highlighted as a potential drug against COVID-19, which currently poses a great threat to our healthcare systems. Metformin leads to reduced blood glucose and improves insulin sensitivity. It has been found that metformin can play an anti-viral role against COVID-19 by inhibiting viral multiplication and the maturation and translation of viral proteins. Because of its anti-inflammatory action, it can modulate the immune response in COVID-19 patients (Gordon et al., 2020; Sharma et al., 2020; Zhu et al., 2020). The proper management of hyperglycemia during COVID-19 infection may also reduce disease severity and decrease the chances of acute respiratory distress syndrome (ARDS). This in turn may help in reducing hospitalizations and deaths among COVID-19 patients with T2DM. Metformin-related activation of AMPK-mediated signaling increases ACE2 receptor phosphorylation and produces a conformational change that may inhibit ACE2–viral spike protein binding, which can eventually hamper viral entry into the cell (Sharmaet al., 2020). Metformin also suppresses the inflammatory response and the release of pro-inflammatory cytokines by inhibiting macrophage activation and NF-κB signaling (Sharma et al., 2020). Additional effects of metformin include antioxidant effects, suppression of host–viral protein interactions (which in turn inhibits host-dependent viral replication, synthesis, and release of viral proteins), and attenuation of endothelial dysfunction, which confers vascular protection and which could help prevent microvascular complications and thrombotic events (Esam 2020; Gordon et al., 2020). Two meta-analyses have shown that metformin administration leads to a significant reduction in COVID-19-associated mortality (Hariyanto and Kurniawan, 2020; Lukito et al., 2020).

## 7 Adverse effects of metformin

Metformin may produce lactic acidosis, a serious condition characterized by severe drowsiness, muscle discomfort, tiredness, chills, blue or cold skin, difficulty breathing, irregular heartbeat, or stomachache with diarrhea (Aldobeaban et al., 2018). Other than this, certain gastrointestinal symptoms with an approximate incidence rate of 20–30% have also been witnessed with use of the drug (Sankhyan and Pawar, 2013; Abdelgadir et al., 2017). Metformin rarely causes hypoglycemia; nevertheless, when used with other anti-diabetic medications, low blood sugar can develop. Increased glucose consumption due to anaerobic metabolism is one probable explanation for metformin-induced hypoglycemia; however, additional processes such as decreased oral intake, decreased liver glucose synthesis, and decreased glucose absorption should also be considered (Aldobeaban et al., 2018). Metformin has a relatively low frequency of producing lactic acidosis in comparison to phenformin (another drug of this class), which makes it the more appropriate and rational choice for use in T2DM (Yendapally et al., 2020) (Table 2).

## 8 Future perspectives

Identified as an anti-malarial, effective against influenza, and a conventional anti-diabetic, metformin’s potential has always appeared to be multi-beneficial (Bailey, 2017), as confirmed by this article’s accounting of the drug’s capability in various activities. The present review outlines various effects of the drug, their respective mechanisms and corresponding results, and the conclusions of several studies confirming the respective precipitating effects. The AMPK pathway has been shown to be the common mechanism underlying metformin’s action. Metformin is associated with few side effects, both in the sense of frequency and severity, and has been observed to be appropriate for a number of conditions, both solely and as an adjuvant, facilitating treatment. It can add substantially to treatment combinations to prevent irreversible and severe stages and enable a faster recovery in critical disorders, including tuberculosis and ocular complications (Maleškić et al., 2017; Han et al., 2018; Xu S et al., 2018; Kusturica et al., 2020). However, there is a need to evaluate metformin in clinical trials before including it in the regime of conventional therapy plans for such critical disorders.
